# Supplementary material for: Somatostatin receptors in fibrotic myocardium
Source: PLoS One. 2024 Jul 22;19(7):e0304813. doi: 10.1371/journal.pone.0304813 (PMC11262693; doi:10.1371/journal.pone.0304813)
Supplement: Attachment — Submitted filename: SSTR paper revisons response - CC02.docx [file pone.0304813.s002.docx]

**PLOS ONE**

Dear Dr. Lawal,

First, we would like to thank Dr. Lawal and the Reviewers for their careful and thoughtful comments. We are grateful for the opportunity to address the issues that they raised. We have considered all comments and present detailed point-by-point responses to each issue raised by Reviewers 1 and 2. We have revised our manuscript, including results from additional analyses.

The corresponding changes are marked highlighted in red in the text and response to the reviewers. Again, we are grateful to the reviewers for their very constructive and insightful comments that we believe have led to an improved manuscript. We very much hope that we have satisfied the questions of the Editor(s) and Reviewers and the revised manuscript is acceptable for publication in PLOS ONE.

Sincerely,

**Response to reviewers**

**“Somatostatin receptors in fibrotic myocardium"**

**PONE-D-23-27842**

**Journal requirements:**

1. *“Please ensure that your manuscript meets PLOS ONE's style requirements, including those for file naming”*

**Response:**

The style requirements have been followed in the revised version of the manuscript.

1. *“Please state what role the funders took in the study. ”*

**Response:**

The funders had no role in study design, data collection and analysis, decision to publish, or preparation of the manuscript. This has been specified in the cover letter and funding sources section of the revised manuscript.

2. *“We note that Figure 1, 3, 4, 5, 6, S1 and S2 in your submission contain copyrighted images”*

**Response:**

No copyrighted images are included in the submission, all images were obtained in our laboratories. This has been specified in the cover letter.

**Reviewer: 1**

**Major concerns**

1. *“In order to compare the relative expression of SSTR and make unbiased comparison, the controls group demographic data have to be presented as well. The SSTR may change due to sex and age, please discuss that”*

**Response:**

Unfortunately, the normal cardiac specimens obtained from LiveOn New York did not include demographic or clinical data beyond their suitability for cardiac transplantation, therefore bias related to sex or age in molecular measurements can not be ruled out. This has been stated in the limitations section.

2. *“Only 50% of HF patients have previous MI, do they have similar magnitude in changes of SSTR in compared to patients with no previous MI. Do LVEF and SSTR show positive correlation?”*

**Response:**

There was no correlation between SSTR mRNA and LVEF of ICM patients. The graphs are show below for the reviewer's perusal.

3. *“Case report - its not really fit into the global scope of the MS, the patient had a relative fresh MI, scar/healing process is completed? In addition, inflammation was detected? Similar Suppl Fig 2 is unnecessary to show.”*

**Response:**

The case report and related images have been removed from the new version of the manuscript

4. *“It will be ideally and more supportive if the authors would provide 68Ga-DOTATATE PET/CT data; including IM cardiomyopathy patients. The n numbers are extremely low, this is not enough for proper conclusion and interpretations.”*

**Response:**

According to the previous question (3.) raised by Reviewer 1, the PET/CT data have been removed in the new version of the manuscript, which now focuses on the tissue analysis findings.

5. *“mRNA expression levels, SSTR show no significant changes between the group, but was a large differene in protein expression levels, please discuss that”*

**Response:**

A paragraph discussing these findings has been added to the discussion, which now reads as follows:

“We showed, in a small cohort of ICM patients, that SSTR1 tends to be upregulated at the expression and protein level in end-stage fibrotic myocardium with ongoing cardiac remodeling and stress but without overly active pro-fibrotic remodeling. SSTR2 expression was not altered in ICM patients, but its protein levels were increased. This may be consequence of postranslational processes that lead to sustained increased protein presence despite minor upregulation of gene expression.”

6. *“Figure 4 - SSTR2 seems to be expressed in cardiomyocytes as well, however the authors stated that "We showed, in a small cohort of ICM patients, that SSTR1 tends to be upregulated at the expression and protein level in end-stage fibrotic myocardium with ongoing cardiac remodeling and stress but without overly active pro-fibrotic remodeling". In order to clarify that the investigators should also apply IHC staining for cardiomyocytes”*

**Response:**

The present of SSTR in cardiomyocytes has been previously reported, as stated in the third paragraph of the discussion. We have updated the language to avoid confusion. The section now reads as follows:

“We showed, in a small cohort of ICM patients, that SSTR1 tends to be upregulated at the expression and protein level in end-stage cardiomyopathy myocardium with ongoing cardiac remodeling and stress and present fibrotic scar but without overly active pro-fibrotic remodeling.”

**Reviewer: 2**

1. *“I have questions for the IHC block, which need to be clarified if we want to be sure of the accuracy of the results obtained and their correct interpretation. For a more correct interpretation and to determine the quality of the IHC staining performed and to ensure the reproducibility of the results, the manuscript lacks information about the IHC technique itself, namely the IHC protocol, what buffer solution was used, the imaging system used and the secondary antibodies (avidin-biotin imaging method or other, horseradish peroxidase or alkaline phosphatase, what reagents were used and how long the peroxidase block was performed).”*

**Response:**

The IHC section of the methods has been expanded, and now reads as follows:

“Briefly, sections of paraffin-embedded tissues were deparaffined by 30 min incubation at 70°C and three washes of 100% xylene. Samples were rehydrated in progressively diluted ethanol and subsequently underwent antigen retrieval by incubation in citrate buffer (Sigma) at 60°C. Samples underwent blocking in 3% bovine serum albumin diluted in phosphate-buffer saline. Incubation with primary antibodies was conducted in a humidity chamber overnight at 4°C with the above indicated concentrations diluted in Dako wash buffer (Dako). Quenching of the endogenous peroxidase was performed by incubation of samples for 10 min with 3% H2O2. After 1h incubation with HPR-linked secondary antibodies (Abcam), development was performed by DAB Substrate Kit (Abcam) with subsequent hematoxylin counterstain (Sigma). Stained samples were dehydrated and cleared and mounted with Permount mounting medium (Sigma).”

2. *“In Figure 7, the presented SSTR1, SSTR2 preparations show intense positive staining of cardiomyocytes (CMC), weak staining of fibrosis areas, and intense staining of individual cells in the fibrosis area (presumably macrophages). Based on previously published works (5) (*[*https://pubmed.ncbi.nlm.nih.gov/31197742/*](https://pubmed.ncbi.nlm.nih.gov/31197742/) *), it was shown that the positive expression of SSTR1 and SSTR2 by activated macrophages in the area of sarcoid granulomas is well detected by IHC, while the rest of the heart tissue, in particular, CMC are negative in staining. Positive staining of macrophages by IHC is expected. Intense staining of SMCs is an unexpected result that requires additional verification and caution in interpreting the results.”*

**Response:**

We have incorporated the suggested reference into the discussion, which now reads as follows:

“The presence of SSTR in different cardiac cell populations has been assessed previously, with somewhat discrepant results. Bravo et. al [5] did not detect positive staining of SSTRs outside granuloma areas in hearts with sarcoidosis. Smith et al. explored the presence of SSTR in cardiac cell populations, identifying SSTR in cardiomyocytes and fibroblasts [9]. In cardiac fibroblasts, SST mobilized intracellular calcium [9]. A role for SSTR in cardiac adaptation to stress and injury has also been proposed. Vörös et al. reported cardioprotective effects of SST and SSTR1/2 in cardiac ischemia reperfusion injury [11]. SSTR1,2 and 5 were expressed in healthy human heart, with RNAscope indicating that SSTR1,2 expression occurred in vascular endothelial cells and cardiomyocytes [11]. Interestingly, SST peptide was decreased in human ICM. Therefore, SSTR1,2 upregulation may be a compensatory response to the decrease in SST levels”.

3. *“Nonspecific staining should be ruled out for a more reliable interpretation of the results. One possible reason for nonspecific staining of cardiomyocytes is the use of polyclonal antibodies; the presence of antibodies to multiple epitopes may increase the likelihood of cross-reactivity with other proteins. There is less risk of cross-reactivity when monoclonal antibodies are used. If possible, if paraffin blocks with tissue samples are preserved, it makes sense to make additional slices from the same blocks and stain them with monoclonal antibodies to SSTR1, SSTR2.”*

**Response:**

We have performed staining of additional slices with monoclonal antibodies, obtaining a similar result to the one obtained with polyclonal antibodies. The results are shown below for both SSTR1 and SSTR2 monoclonal staining:

4. *“At least for SSTR2, it makes sense to use pancreatic tissue as an additional control (positive - islets of Langerhans, and negative - the rest of the pancreatic tissue)..”*

**Response:**

We have performed staining of SSTR2 on human pancreatic tissue sample. The results are shown below and included in the new version of figure 3.

5. *“Another possible reason for nonspecific staining of cardiomyocytes when using the immunoperoxidase staining system is insufficient inhibition of endogenous peroxidases. All samples containing hemoproteins, including myoglobin in muscle cells, hemoglobin in erythrocytes, and cytochrome in granulocytes and monocytes, may show unsuppressed endogenous peroxidase activity. There is experimental evidence that myoglobin concentration in cardiomyocytes may increase during prolonged hypoxia (*[*https://pubmed.ncbi.nlm.nih.gov/19005161/*](https://pubmed.ncbi.nlm.nih.gov/19005161/) *;* [*https://pubmed.ncbi.nlm.nih.gov/20675543/*](https://pubmed.ncbi.nlm.nih.gov/20675543/) *).Intense positive staining of cardiomyocytes appeared in patients with severe heart failure, it is known that tissues of such patients suffer from hypoperfusion and experience hypoxia, which, theoretically, can lead to increased expression of myoglobin and increased activity of endogenous peroxidases. To eliminate the influence of this factor in IHC staining, change the peroxidase block or increase the tissue incubation time with it or choose a different tagging enzyme such as alkaline phosphatase..”*

**Response:**

Our IHC protocol, now detailed in the methods, includes quenching of the endogenous peroxidase. If myoglobin were influencing staining in ICM samples, the same effect would appear in the samples stained with SSTR5, included in figure 4.

6. *“The above factors may change the answer to the main question: What exactly is the tissue substrate for PET imaging when using somatostatin-directed radiopharmaceuticals (68Ga-DOTATATE)? Previous work suggests that this substrate is very likely to be inflammatory cells, particularly macrophages. This work claims to identify a fundamentally new substrate - directly the SMCs themselves, under certain conditions (terminal CHF). Since this work claims to validate fundamentally new knowledge, this requires additional careful scrutiny to avoid unpleasant mistakes. This is the basis for my recommendations.”*

**Response:**
Since upregulation of SSTRs at the protein level is possible in cardiac areas without active inflammation, it is possible that 68Ga-DOTATATE can make myocardial areas that are not inflamed visible by PET imaging.

**Minor Issues**

7. *“If the mean mRNA content of SSTR1 and SSTR2 did not differ between the study group and the control group, how to explain this difference with respect to the IHC pattern, where differences in staining intensity are clearly visible?”*

**Response:**

A paragraph discussing these findings has been added to the discussion, which now reads as follows:

“We showed, in a small cohort of ICM patients, that SSTR1 tends to be upregulated at the expression and protein level in end-stage fibrotic myocardium with ongoing cardiac remodeling and stress but without overly active pro-fibrotic remodeling. SSTR2 expression was not altered in ICM patients, but its protein levels were increased. This may be consequence of postranslational processes that lead to sustained increased protein presence despite minor upregulation of gene expression.”

8. *“A clinical case of a patient with a combination of NEO and ischemic cardiopathy is given as background. Unfortunately, I could not understand from the text of the article whether any of the patients in the main group (n=8, with ischemic cardiopathy) was diagnosed with HEO, or whether all patients in the main group were without HEO? I think this point is important; it may be better to spell it out more clearly. If the patients were without NEO, what were the indications for PET with 68Ga-DOTATATE?”*

**Response:**

Following recommendations from reviewer 1, the case report has been removed from the current version of the manuscript.

9. *“Numbering of figures is confused - Figure 1 in the text corresponds to Figure 2 in the appendix, Figure 2 in the text corresponds to Figure 3 in the appendix, etc.*

**Response:**

This has been fixed.
